# General Ability Level Moderates Cognitive–Achievement Relations for Mathematics

**DOI:** 10.3390/jintelligence13060065

**Published:** 2025-06-05

**Authors:** Christopher R. Niileksela, Jacob Robbins, Daniel B. Hajovsky

**Affiliations:** 1 Department of Educational Psychology, University of Kansas, Lawrence, KS 66045, USA; 2 Department of Educational Psychology, Texas A&M University, College Station, TX 77845, USA; jarobbins93@tamu.edu (J.R.); dhajovsky@tamu.edu (D.B.H.)

**Keywords:** cognitive ability, academic skills, cognitive–achievement relations, Spearman’s Law of Diminishing Returns

## Abstract

Spearman’s Law of Diminishing Returns (SLODR) suggests general intelligence would be a stronger predictor of academic skills at lower general ability levels, and broad cognitive abilities would be stronger predictors of academic skills at higher general ability levels. Few studies have examined how cognitive–mathematics relations may vary for people with different levels of general cognitive ability. Multi-group structural equation modeling tested whether cognitive–mathematics relations differed by general ability levels for school-aged children (grades 1–5 and grades 6–12) using the *Woodcock-Johnson Third Edition* (*n* = 4470) and *Fourth Edition* (*n* = 3891) standardization samples. Results suggested that relationships between cognitive abilities and mathematics varied across general ability groups. General intelligence showed a stronger relative effect on mathematics for those with lower general ability compared to those with average or high general ability, and broad cognitive abilities showed a stronger relative effect on mathematics for those with average or high general ability compared to those with lower general ability. These findings provide a more nuanced understanding of cognitive–mathematics relations.

## 1. Introduction

“*In the learning environment, intelligence becomes a force of transformation, not just understanding*.”

Arthur Costa

We rely on our ability to learn to function successfully in the world. Our capacity to acquire, construct, and apply knowledge affects our physical health, mental health, and success in school and work (Kuncel and Hezlett 2010; Wrulich et al. 2014). Understanding the factors that are related to how we learn and acquire academic skills is an important line of inquiry. Understanding those factors may help us improve our approaches to teaching, intervention, and evaluation. For instance, general cognitive ability (i.e., general intelligence) has been found to account for 50–70% of the variation in academic skills (Deary et al. 2007; Kaufman et al. 2012), with more specific cognitive abilities (e.g., fluid reasoning, verbal comprehension) accounting for a smaller, yet not unsubstantial, proportion of variance beyond general cognitive ability (e.g., Zaboski et al. 2018). By cognitive abilities, we are referring to those abilities such as working memory, reasoning, and visual-spatial abilities that are less influenced by direct instruction and exposure during formal schooling. By academic skills we are referring to reading, writing, or mathematics skills that are typically learned through direct instruction and exposure during formal schooling (Schneider and McGrew 2018). General and specific cognitive abilities are an important component of academic skill acquisition, and research suggests that cognitive development promotes academic success in children (Rinaldi and Karmiloff-Smith 2017; Stockard et al. 2018).

The current study focuses on the relationships between cognitive abilities and mathematics skills. The influence of cognitive abilities on academic skills has been shown to vary across variables such as age and achievement domain (e.g., reading vs. mathematics; Caemmerer et al. 2023; Peng and Kievit 2020). Research on these cognitive–achievement relations and the variables that moderate those relations can help researchers and practitioners develop a more comprehensive understanding of how learning occurs and what variables predict achievement for different individuals. The current study examines whether there are differences in cognitive–achievement relations for people with different levels of general cognitive ability. Although some work in this area has been completed as it relates to reading (e.g., Caemmerer et al. 2024; Hajovsky et al. 2023), to our knowledge no studies have examined how cognitive–achievement relations may differ across the general cognitive ability spectrum for mathematics. This study aims to explore differences in cognitive–achievement relations for mathematics at different levels of general cognitive ability to determine if and to what degree these relations differ across the general cognitive ability spectrum.

### 1.1. Importance of Mathematics Skills

Mathematics skills are important in various contexts across the lifespan. In early childhood, mathematics skills are foundational for understanding numbers and basic arithmetic, fostering cognitive development. As children progress through school, mathematics skills become essential for success in science, technology, engineering, and mathematics (STEM) fields. In middle adulthood, mathematics skills remain valuable in problem solving, financial planning, and data analysis, aiding career advancement and informed decision-making (Duckworth et al. 2012; Jansen et al. 2016; Reyna and Brainerd 2007). Mathematics skills are an important asset that may help guide individuals through the challenges and opportunities of life’s stages. As such, it may be helpful to understand which cognitive abilities are related to mathematics for people with different levels of general cognitive ability. If the cognitive abilities that predict mathematics differ for people with higher and lower general cognitive ability, that can be helpful information for understanding the development of mathematics, instructional planning, and it can add to the theoretical base about mathematics development.

### 1.2. Cattell–Horn–Carroll (CHC) Theory of Intelligence

This study is guided by the Cattell–Horn–Carroll (CHC) theory of intelligence, which suggests that intelligence is multidimensional and composed of distinct abilities that form an integrated system of problem-solving skills and a fund of cultural knowledge (Carroll 1993; Schneider and McGrew 2018). In CHC theory, cognitive abilities are hierarchically structured, with each descending level representing more specialized or domain-specific abilities. At the topmost level is general cognitive ability, or *g*, which represents overall cognitive functioning and influences several broad cognitive abilities. Below *g* and appearing at the second level are approximately eight broad cognitive abilities that typically include comprehension–knowledge (G*c*), fluid reasoning (G*f*), working memory (G*wm*), learning efficiency (G*l*), retrieval fluency (G*r*), visual processing (G*v*), auditory processing (G*a*), and processing speed (G*s*). Below each broad cognitive ability and appearing at the third level are various narrow cognitive abilities, of which more than 80 have been identified (e.g., associative memory, lexical knowledge, deduction; Schneider and McGrew 2018). CHC is a continually evolving taxonomy that has been updated with current research. For example, G*l* and G*r* used to be included under a single broad cognitive ability called long-term retrieval (G*lr*), but evidence suggested that G*l* and G*r* represented distinct broad cognitive abilities rather than narrow cognitive abilities under a single broad cognitive ability (Schneider and McGrew 2018). CHC theory is a useful taxonomy for organizing research findings, understanding which cognitive abilities influence learning outcomes, and how much they influence learning outcomes. In this study, both *g* and the broad cognitive abilities are examined to understand how both individually and collectively are related to mathematics for those with differing levels of general cognitive ability, or *g*.

### 1.3. Cognitive–Achievement Relations in Mathematics

Mathematics skills are typically broken down into calculation (e.g., solving decontextualized mathematics problems) and mathematics problem solving (e.g., solving applied mathematics problems and that require the application of mathematics concepts in realistic situations; McGrew and Wendling 2010). McGrew and Wendling (2010) synthesized research on cognitive–achievement relationships for mathematics and found that G*f*, G*c*, G*s*, and G*wm* predicted both calculation and mathematics problem solving across multiple studies. On the other hand, G*a*, G*v*, and G*lr* did not consistently predict calculation and mathematics problem solving. More recent studies support these results (e.g., Cormier et al. 2017; Niileksela et al. 2016). Zaboski et al. (2018) completed a meta-analysis of published studies on cognitive–achievement relations and found that *g* accounted for much of the variation in mathematics skills, although G*f*, G*c*, and G*s* were also related to mathematics.

Most cognitive–achievement relations research on mathematics has focused on how cognitive abilities predict calculation and math problem solving separately. However, little cognitive–achievement relations research has examined how these two mathematics skills may be integrated and influence each other. For example, it is possible that cognitive abilities predict calculation, and then both cognitive abilities and calculation predict math problem solving. The assumption of these models is that the link between cognitive ability and advanced academic skills (i.e., math problem solving) is partially mediated through basic academic skills (i.e., calculation; Hajovsky et al. 2023). This is important to consider because it is possible that the development of calculation is assumed to be an important precursor and predictor to the development of math problem solving. Both general and broad cognitive abilities have been shown to be related to math problem solving via calculation (Decker and Roberts 2015; Villeneuve et al. 2019), and research has supported this finding for the relationship between basic reading skills and reading comprehension (e.g., Floyd et al. 2012). This integrated model of cognitive–achievement relations for mathematics is used in the current study.

### 1.4. Cognitive–Achievement Relations Across Ability Levels

Most previous research on cognitive–achievement relations focused on normative samples, so the research findings with these samples represent the average magnitude of these relationships for individuals across the general cognitive ability spectrum. It remains unclear how cognitive–achievement relations may vary across different general cognitive ability levels. Some work seems to support Spearman’s Law of Diminishing Returns (SLODR) in cognitive–achievement relations, where broad cognitive abilities are stronger predictors of achievement in higher-ability individuals and general ability is a stronger predictor of achievement in lower-ability individuals (Breit and Preckel 2020; McLarnon et al. 2018, respectively). However, recent work suggests that both the directionality and magnitude of relation between cognitive abilities and achievement may depend on the type of ability or academic domain (Schmitt et al. 2017; Peng and Kievit 2020). Other theoretical frameworks also suggest this finding. For example, mutualism (Peng and Kievit 2020) claims that academic skills and cognitive abilities become increasingly supportive of one another across development (van der Maas et al. 2006). Thus, mutualistic factors like gene–environment interactions, schooling, and learning opportunities can either maximize or hinder the degree to which cognitive ability and academic skills bestow their respective influences (Peng and Kievit 2020).

SLODR also suggests that the relationships among cognitive abilities differ based on general ability level. SLODR asserts that, as levels of *g* increase, its structure (broad and specific abilities) becomes more differentiated, where *g* has a weaker influence on cognitive performance and broad or specific abilities have stronger influences on cognitive performance (Murray et al. 2013). In contrast, as levels of *g* decrease, *g* has a stronger influence on cognitive performance and broad or specific abilities have a weaker influence on cognitive performance. In other words, *g* accounts for less variation in broad cognitive ability performance when *g* is higher, resulting in more differentiation among broad cognitive abilities (i.e., weaker correlations; Hajovsky et al. 2023). As such, it would be predicted that the association between broad cognitive abilities and achievement would be stronger for those with higher ability (Hajovsky et al. 2023; McLarnon et al. 2018), whereas the association between *g* and achievement would be stronger for those with lower ability. Theoretical insights from SLODR and mutualism suggest cognitive–achievement relations would vary for different ability levels. These findings are important because they suggest that the relationships among cognitive abilities and academic skills are not necessarily the same across people, and individual differences in multiple variables may moderate the strength of the relationship between cognitive abilities and academic skills.

### 1.5. Current Study

Previous work has not examined whether cognitive–achievement relations for mathematics differ across the general ability spectrum. Although some research has examined differences in cognitive–achievement relations across the spectrum of academic skills spectrum rather than cognitive ability (Hajovsky et al. 2020), this previous work examined differences in relations for the dependent variable, not the independent variable. The current study examines whether cognitive–achievement relations differ at the level of an independent variable, which in this case is general cognitive ability for school-aged children (grades 1–5 and grades 6–12) using the *Woodcock-Johnson Third Edition* and *Fourth Edition*. This study uses the same test batteries and methodology as Hajovsky et al. (2023), which examined differences in cognitive–achievement relations for reading across the general cognitive ability spectrum. This study further expands the cognitive–achievement literature by investigating whether and how cognitive–achievement relations for mathematics skills differ across the general cognitive ability spectrum with similar measures and methods as previous work (Hajovsky et al. 2023).

The current study is designed to examine whether relationships between cognitive abilities and mathematics differ based on general ability level in two different national standardization samples. The current study builds on previous research by examining ability-based changes in cognitive–achievement relationships in the domain of mathematics. Based on previous research and theoretical expectations, it is predicted that cognitive–achievement relationships will vary for different general cognitive ability levels. In line with SLODR, it is expected that the relationship between general cognitive ability and mathematics skills will be stronger for those with lower levels of general cognitive ability, and the relationships between broad cognitive abilities and mathematics skills will be stronger for those with high levels of general cognitive ability.

## 2. Materials and Methods

### 2.1. Participants

The data for this study included school-age children and adolescents from the standardization samples for the *Woodcock-Johnson Third Edition* (WJ III, Woodcock et al. 2001, 2007) and *Woodcock-Johnson Fourth Edition* (WJ IV, (Schrank et al. 2014) tests of cognitive abilities and achievement. These are the same samples used in previous research examining differences in cognitive–achievement relations across the general ability spectrum (Hajovsky et al. 2023). The WJ III and WJ IV were both analyzed to determine if findings were similar across these two test batteries and samples. The standardization samples for the WJ III and WJ IV represented the U.S. population at the time of standardization, where the sample was stratified on race/ethnicity, sex, country of birth, community type, census region, parent education, school type, occupational level, and employment status (McGrew et al. 2007, 2014). There were 4470 children and adolescents in kindergarten through 12th grade in the WJ III sample and 3891 children and adolescents in kindergarten through 12th grade in the WJ IV sample. The WJ III and WJ IV samples were divided into elementary (1st–5th grade) and secondary (6th–12th grade) groups. Previous research suggests there are developmental differences in the relations between cognitive abilities and mathematics skills (e.g., Cormier et al. 2017; Floyd et al. 2003; Niileksela et al. 2016), so dividing the samples in this way would help account for these potential differences. The data are not publicly available and were used with permission from the Woodcock Institute for the Advancement of Neurocognitive Research and Applied Practice. Given the secondary analytical approach of this study, informed consent was not required. The study was reviewed by a university institutional review board and was deemed to not be human subjects research. This research did not receive any specific grants from funding agencies in the public, commercial, or not-for-profit sectors.

### 2.2. Measures

Composite scores from the WJ III and WJ IV were used for all analyses. All scores are age-based standard scores, with means of 100 and standard deviations of 15. Composite scores were designed to measure cognitive and academic skills based on CHC theory (Schneider and McGrew 2018). Cognitive composites included General Intellectual Ability (GIA) and seven broad cognitive abilities, which were Comprehension–Knowledge (G*c*), Fluid Reasoning (G*f*), Short-Term Working Memory (G*wm*), Processing Speed, (G*s*), Long-Term Storage and Retrieval (G*lr*), Visual Processing (G*v*), and Auditory Processing (G*a*). Achievement composites included two mathematics skills, Math Calculation (MC) and Math Problem Solving (MPS). The technical manuals for the WJ III (McGrew et al. 2007) and WJ IV (McGrew et al. 2014) provide validity and reliability evidence for the composite scores.

#### 2.2.1. Math Calculation

The Math Calculation (MC) composite provides a measure of an individual’s ability in calculation skills, including basic mathematics through calculus. On both the WJ III and WJ IV the composite includes two tests: Calculation (examinees complete increasingly difficult calculation problems) and Math Fact Fluency (examinees complete as many single-digit math facts using addition, subtraction, and multiplication in three minutes). Reliability for MC on the WJ III ranged from 0.87 to 0.93 across ages 5–19 (McGrew et al. 2007) and on the WJ IV ranged from 0.80 to 0.90 across ages 5–19 (McGrew et al. 2014).

#### 2.2.2. Math Problem Solving

The Math Problem Solving (MPS) composite provides a measure of an individual’s ability to apply mathematics skills in real-world situations. On the WJ III the composite includes two tests: Applied Problems (examinees complete increasingly difficult word problems that require application of mathematics skills) and Quantitative Concepts (examinees complete two shorter tasks, including Number Series and Concepts that require reasoning through problems with numbers and demonstrating knowledge of mathematics concepts and vocabulary). On the WJ IV, this composite includes Applied Problems (same as the corresponding test on the WJ III) and Number Matrices (examinees provide a number that completes a matrix of numbers). Reliability for MPS on the WJ III ranged from 0.80 to 0.90 across ages 5–19 (McGrew et al. 2007) and on the WJ IV ranged from 0.81 to 0.91 across ages 5–19 (McGrew et al. 2014).

#### 2.2.3. General Intellectual Ability

The General Intellectual Ability (GIA) composite is an estimate of psychometric *g*. On both the WJ III and WJ IV, the GIA includes seven tests, one each from the seven broad cognitive abilities measured by the WJ Batteries. The GIA on the WJ III includes Verbal Comprehension, Concept Formation, Sound Blending, Spatial Relations, Visual-Auditory Learning, Visual Matching, and Numbers Reversed. The GIA on the WJ IV includes Oral Comprehension, Number Series, Verbal Attention, Letter Pattern Matching, Phonological Processing, Story Recall, and Visualization. Reliability estimates for the GIA ranged from 0.96 to 0.97 across ages 5–19 on the WJ III (McGrew et al. 2007) and 0.95 to 0.97 across ages 5–19 on the WJ IV (McGrew et al. 2014).

#### 2.2.4. Broad CHC Cognitive Abilities

Composite scores that represent the seven broad cognitive abilities included on the WJ III and WJ IV were used. Composite scores for the broad cognitive abilities were estimated using two tests that measure different narrow cognitive abilities that are subsumed under the broad cognitive ability. The WJ III and WJ IV used slightly different tests for the broad cognitive composite scores. Table 1 includes a description of the composites and the names of the tests that comprise each of the broad cognitive ability composites. Reliability coefficients for the WJ III broad cognitive ability composites ranged between 0.70 and 0.96 for school-aged children and adolescents (ages 5–19, Woodcock et al. 2001, 2007) and reliability coefficients for the WJ IV broad cognitive ability composites ranged between 0.80 and 0.98 for school-aged children and adolescents (McGrew et al. 2014).

### 2.3. Data Analytic Plan

#### Identifying Ability Groups

To test whether relations between cognitive abilities and mathematics differed across general cognitive ability groups, different ability groups had to be identified. The methods used to identify different ability groups were the same as those used in Hajovsky et al. (2023). These groups included individuals with general cognitive ability levels in the low (<25th percentile), average (25th–75th percentile), and high (>75th percentile) ranges. The 25th percentile is commonly used as a point suggesting that cognitive or academic skills may require follow-up or further scrutiny when conducting evaluations (e.g., Fletcher et al. 2019). Although using more extreme percentiles (e.g., 10th percentile for low, 90th percentile for high) may accentuate differences across the ability groups, the sample sizes for the low and high ends of the general ability spectrum would have been much smaller, resulting in a substantial loss of power to detect differences in cognitive–achievement relations across groups. Thus, the use of the 25th and 75th percentiles ensured that sample sizes for each of the ability groups would have sufficient power to detect effects in the analyses.

Selecting groups using variables that are included in the analysis is statistically problematic because it can result in a restriction of range, which can affect the magnitude of the relationships among variables in the analysis. Because the primary purpose of this study was to examine relationships between cognitive and mathematics skills, reducing these effects from selection was important to consider. In this study, an alternative estimate of general intellectual ability (altGIA) was used to select general cognitive ability groups. The altGIA was estimated using seven supplemental tests from the WJ III and WJ IV that measure each of the seven broad CHC cognitive abilities. None of the tests included in the altGIA were used in composites included in other analyses in this study. In other words, none of the measures used to identify the general cognitive ability groups were used in any of the primary analyses of the study to reduce the statistical issues related to the data analysis on the variables used to select groups.

The altGIA for the WJ III samples included Picture Vocabulary (G*c*), Memory for Names (G*lr*), Block Rotation (G*v*), Incomplete Words (G*a*), Number Series (G*f*), Cross Out (G*s*), and Memory for Sentences (G*wm*). The altGIA for the WJ IV samples included Picture Vocabulary (G*c*), Analysis-Synthesis (G*f*), Number Pattern Matching (G*s*), Memory for Words (G*wm*), Sound Blending (G*a*), Memory for Names (G*lr*), and Visual Closure (G*v*). Confirmatory factor analysis was used to estimate a one-factor model using these tests for the WJ III and WJ IV separately. This one-factor model represented a general intelligence factor. A factor score was estimated for all participants, and then those factor scores were used to identify the low (<25th percentile), average (25th–75th percentile), and high (>75th percentile) general cognitive ability groups. The one-factor model and factor scores were estimated separately for the elementary and secondary samples of the WJ III and WJ IV.

The validity of the altGIA as an estimate of general intelligence that could be used to select ability groups was examined by correlating the latent factor of the altGIA for the WJ III and WJ IV with the latent factor that included the tests for the GIAs on the WJ III and WJ IV, respectively. The correlation between these latent factors was 0.99 for the WJ III elementary and secondary samples, and the correlation between these latent factors was 1.00 for the WJ IV elementary and secondary samples. The latent estimates of the GIA and altGIA were essentially equivalent. Reliability of the altGIA was estimated using coefficient omega. In the WJ III samples omega was 0.69 and 0.74 for the elementary and secondary samples, respectively, and in the WJ IV samples omega was 0.69 and 0.70 for the elementary and secondary samples, respectively. All omega values suggest adequate reliability of the altGIA as an estimate of general intelligence.

### 2.4. Integrated Cognitive–Achievement Models

Similarly to Hajovsky et al. (2023), multi-group path analysis and structural equation modeling were used to simultaneously estimate models for the low-, average-, and high-ability groups. The magnitude of regression coefficients was compared across groups by adding cross-group equality constraints to corresponding regression parameters across groups. When cross-group equality constraints were added to models, the likelihood ratio test was used to determine if there was a statistically significant degradation in model fit, which would suggest there are differences in the size of the regression coefficients across groups. A non-statistically significant degradation in model fit would suggest no differences in the size of the regression coefficients across groups.

**Figure 1:**
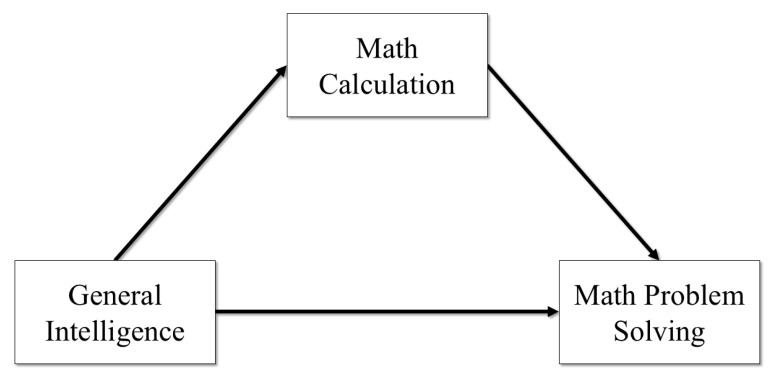
Integrated achievement model for general intelligence predicting mathematics.

**Figure 2:**
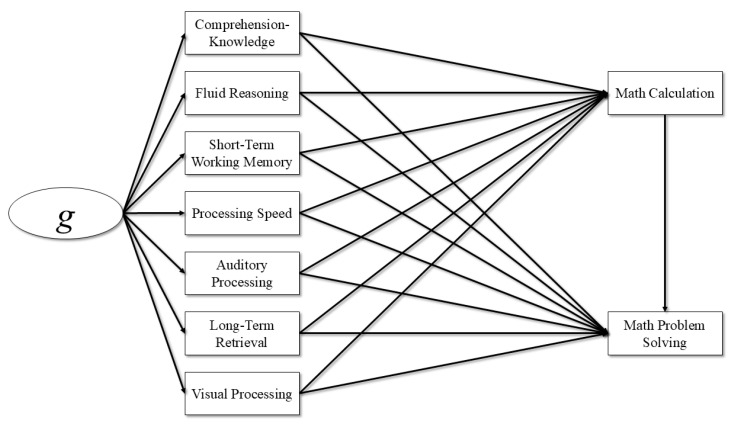
Integrated achievement model for broad cognitive abilities predicting mathematics.

Figure 1 and Figure 2 show each of the integrated cognitive–achievement models that were estimated for this study. In the integrated cognitive–achievement relations models, cognitive abilities were predictors of both Math Calculation and Math Problem Solving, and then Math Calculation was a predictor of Math Problem Solving. This model assumes that cognitive abilities predict performance on both basic and complex academic skills, and basic academic skills predict performance on complex academic skills. In these models, the effects of cognitive abilities on complex academic skills are assumed to be partially mediated though basic academic skills. In the first model, Math Calculation was regressed on the GIA and Math Problem Solving was regressed on both the GIA and Math Calculation. This model was estimated to examine differences in the effects of general intelligence on math skills across general cognitive ability groups. In the second model, Math Calculation was regressed on the broad cognitive abilities and Math Problem Solving was regressed on the seven broad cognitive abilities and Math Calculation. This model was estimated to examine differences in the effects of the seven broad cognitive abilities on math skills across ability groups. This model also included a latent *g* factor that was measured by the seven broad cognitive abilities. This model allowed for the estimation of the effects of general intelligence and the broad cognitive abilities on math skills across ability groups. Although the effects of general intelligence on math skills are indirect, the variance accounted for in Math Calculation and Math Problem Solving and Math Calculation due to general intelligence and the broad cognitive abilities can be separated to examine any differences across groups.

### 2.5. Analytical Software and Missing Data

M*plus* 7.4 (Muthén and Muthén 1998–2015) was used for all analyses. All missing data were assumed to be missing at random (MAR) because not all tests were administered to every person in the standardization sample (i.e., the standardization samples used a planned missing data design, McGrew et al. 2014), so scores were not likely to be missing because of the individual’s ability on variables with missing scores (Enders 2022). All models were estimated using full-information maximum likelihood (FIML) and all cases were included in the analysis.

## 3. Results

### 3.1. Descriptive Statistics

Descriptive statistics for all WJ III and WJ IV test scores across ability groups are in Table 2 and Table 3. Means for all scores were in ranges that would be expected based on the selection of groups using the altGIA, where means for the low group were in the 80s, means for the average group were near 100, and means for the high group were in the 110s. Standard deviations were between 10 and 15 for most scores across groups. Although standard deviations for the GIA were smaller, this was expected because groups were selected using the altGIA, which was highly correlated with the GIA. All scores were normally distributed, with skewness values < |2| and kurtosis values < |7|, suggesting that the use of maximum likelihood estimation was appropriate (Curran et al. 1996).

Table 4 includes unstandardized and standardized coefficients across the WJ III and WJ IV elementary and secondary samples. The *R*^2^ for Math Calculation and Math Problem Solving for each ability group and sample is included. Pairwise comparisons of the regression coefficients across the low, average, and high groups for all paths in the model were examined to determine where statistically significant differences in path coefficients occurred. For the WJ III elementary sample, the path from the GIA to Math Calculation was higher for the low group (β = 0.48) compared to the average (β = 0.27) and high (β = 0.28) groups. The path from Math Calculation to Math Problem Solving was higher for the low (β = 0.49) and high (β = 0.42) groups compared to the average group (β = 0.33). The path from the GIA to Math Problem Solving was statically equal across groups.

In the WJ III secondary sample, the path from the GIA to Math Calculation was higher for the low group (β = 0.43) compared to the average (β = 0.23) and high (β = 0.30) groups. The path from Math Calculation to Math Problem Solving was higher for the high group (β = 0.52) compared to the average group (β = 0.42). The path from the GIA to Math Problem Solving was higher for the low group (β = 0.36) compared to the average (β = 0.19) and high (β = 0.21) groups.

For both the WJ III elementary and secondary samples, the low group had larger *R*^2^ values for Math Calculation and Math Problem Solving compared to the average and high groups. Although the size of the regression paths was not statistically significantly different across groups for the WJ IV elementary and secondary samples, *R*^2^ values were larger for Math Calculation in the low group compared to the average and high groups.

#### 3.1.1. Broad Cognitive Abilities and Indirect Effects of *g* Predicting Mathematics

In the second model, Math Calculation was regressed on the broad cognitive abilities and Math Problem Solving was regressed on Math Calculation and the broad cognitive abilities. The broad cognitive abilities loaded on a single factor, representing *g*. There was not a direct path from *g* to Math Calculation and Math Problem Solving, but the indirect effects of *g* on the two mathematics composites were estimated. Overall, model fit was marginal for these analyses based on conventional standards for fit indexes (e.g., Schermelleh-Engel et al. 2003). Values for the root mean square error of approximation (RMSEA) and standardized root mean square residual (SRMR) suggested adequate model fit (RMSEA < 0.07, SRMR < 0.09). However, values for the comparative fit index (CFI) and Tucker–Lewis index (TLI) suggested marginal model fit (values between 0.90 and 0.95). This was the case for the WJ III elementary sample (CFI = 0.911, TLI = 0.854, RMSEA = 0.040, and SRMR = 0.058), WJ III secondary sample (CFI = 0.946, TLI = 0.911, RMSEA = 0.040, and SRMR = 0.061), WJ IV elementary sample (CFI = 0.917, TLI = 0.864, RMSEA = 0.072, and SRMR = 0.063), and WJ IV secondary sample (CFI = 0.920, TLI = 0.868, RMSEA = 0.069, and SRMR = 0.057). When cross-group equality constraints were added to the paths from the broad cognitive abilities to mathematics there was not a statistically significant degradation in model fit for the WJ III elementary sample, χ^2^ (30) = 34.06, *p* = .278, and for the WJ IV secondary sample, χ^2^ (30) = 31.09, *p* = .411. There was a statistically significant degradation in model fit for the WJ III secondary sample, χ^2^ (30) = 62.17, *p* < .001, and for the WJ IV elementary sample, χ^2^ (30) = 43.92, *p* = .049, suggesting differences in the size of the path coefficients across the different ability groups for those samples.

Table 5 and Table 6 include unstandardized and standardized coefficients across the WJ III and WJ IV elementary and secondary samples. The *R*^2^ for Math Calculation and Math Problem Solving for each ability group and sample is included. Pairwise comparisons across the low, average, and high groups for all paths in the model were examined to determine where there were statistically significant differences in path coefficients. Overall, in the WJ III elementary sample the statistically significant predictors of Math Problem Solving included Math Calculation, G*c*, G*f*, G*s*, and G*wm* and statistically significant predictors of Math Calculation included G*c*, G*f*, G*s*, and G*wm*. The indirect effects of *g* on Math Calculation and Math Problem Solving were also statistically significant across groups. In the WJ III secondary sample, the results were similar to those of the WJ III elementary sample, although there appeared to be some suppressor effects of G*lr*, G*a*, and G*s*, which had significant negative effects on Math Calculation and Math Problem Solving for several groups. For the WJ IV elementary sample, significant predictors of Math Problem Solving included Math Calculation, G*c*, G*v*, G*f*, G*s*, and G*wm*. Significant predictors of Math Calculation were G*c*, G*f*, G*s*, and G*wm*, and there were also some suppressor effects of G*lr* and G*a* in this sample as well. The indirect effects of *g* on mathematics were significant across groups. In the WJ IV secondary sample, statistically significant predictors of Math Problem Solving included Math Calculation, G*c*, and G*f*, and statistically significant predictors of Math Calculation included G*c*, G*f*, G*s* and G*wm*.

For the WJ III secondary sample, the path from Math Calculation to Math Problem Solving was lower for the low (β = 0.48) and average (β = 0.44) groups compared to the high group (β = 0.53). The path from G*lr* to Math Problem Solving was lower for the low group (β = −0.14) compared to the average group (β = 0.03). The path from G*s* to Math Problem Solving was higher for the low group (β = 0.04) compared to the average group (β = −0.11) and the high group (β = −0.09). The path from G*wm* to Math Calculation was higher for the low group (β = 0.19) compared to the high group (β = 0.03). For the WJ IV elementary sample, the path from G*a* to Math Calculation was lower for the low group (β = −0.15) compared to the average group (β = −0.07). The path from G*f* to Math Calculation was higher for the low group (β = 0.46) compared to the average (β = 0.36) and high (β = 0.29) groups.

The indirect effect of *g* on Math Calculation was generally larger for the low group than for the average and high groups. Although 95% confidence intervals overlapped for all unstandardized indirect effects of *g* on Math Calculation and Math Problem Solving, the standardized effects differed across groups, likely because the residual variances for Math Calculation and Math Problem Solving were not equal across groups. In other words, the unstandardized slope of the indirect effect of *g* on Math Calculation and Math Problem Solving was similar across groups, but the variance accounted for by the indirect effect of *g* on Math Calculation and Math Problem Solving differed across groups.

Similar to the results that only included the GIA as a predictor of Math Calculation and Math Problem Solving, the *R*^2^ values were larger for the low group compared to the average and high group across all samples, indicating that cognitive abilities tended to explain more variance in the mathematics skills for the low group compared to the average and high groups.

#### 3.1.2. Relative Effects of Broad Cognitive Abilities and General Intelligence on Mathematics

One observation from the previous analyses was that the *R*^2^ for Math Calculation and Math Problem Solving tended to differ across groups. A more nuanced analysis of the effects of *g* and the effects of the broad cognitive abilities was conducted to examine how the relative amount of variance in Math Calculation and Math Problem Solving was explained separately by *g* and the broad cognitive abilities across ability groups. The methods used to examine the relative effects of *g* and broad cognitive abilities used in Hajovsky et al. (2023) were used here. First, the square of the total standardized indirect effect of *g* on Math Calculation and Math Problem Solving was calculated to represent the variance accounted for by *g* in these two variables. This value was subtracted from the *R*^2^ for Math Calculation and Math Problem Solving to represent the variance accounted for in these two variables by the broad cognitive abilities. Next, the square root of the variance accounted for by broad cognitive abilities was calculated to obtain the total unique effect of the broad cognitive abilities on Math Calculation and Math Problem Solving. Finally, the relative proportion of the *R*^2^ for Math Calculation and Math Problem Solving was calculated to examine how these differed across groups. These results are in Table 7 and Table 8.

Overall, the proportion of variance in Math Calculation and Math Problem Solving that was accounted for by *g* was relatively larger than the proportion of variance accounted for by the broad cognitive abilities for the low group compared to the average and high groups. Across all samples, the relative variance accounted for by *g* tended to be around 30–40% for the low group, but between 10% and 20% for the average and high groups. The broad cognitive abilities accounted for about 60–70% of the relative variance in mathematics for the low group, and between 65% and 90% of the relative variance in the average and high groups. In other words, when examining how much *g* and broad cognitive abilities accounted for variability in mathematics relative to the amount of variance accounted for, *g* had relatively more explanatory power than the broad cognitive abilities for the low group compared to the average and high groups, whereas the broad cognitive abilities had relatively more explanatory power than *g* for the average and high groups compared to the low group. This is consistent with the predictions of SLODR.

## 4. Discussion

This study investigated the differential influence of cognitive abilities on mathematics skills across different levels of general cognitive ability using two large, nationally representative samples of school-age children and adolescents that have been used in previous research examining reading (Hajovsky et al. 2023). In line with past findings, GIA and broad cognitive abilities both predicted math achievement. When the latent construct *g* was extracted from broad cognitive abilities, the broad cognitive abilities explained relatively more variance in mathematics skills for high-ability groups while *g* explained relatively more variance in mathematics skills for the low-ability groups.

### 4.1. Cognitive–Achievement Relations and SLODR

The current results are consistent with previous work indicating that general intellectual ability is a strong predictor of mathematics skills (e.g., Villeneuve et al. 2019; Zaboski et al. 2018). Regarding the WJ III sample, we found that GIA was more predictive of Math Calculation and Math Problem Solving for individuals in the low group compared to the average and high groups. Taken with previous findings (McGill 2015), these results support the notion that the GIA has more pronounced effects on mathematics skills for students with lower levels of general cognitive ability compared to those with average and high levels of cognitive ability. Interestingly, these results were not observed for the WJ IV samples, where there were no differences between ability groups. This discrepancy could be due to differences in the tests that comprise the GIA for the WJ III and WJ IV. Specifically, the WJ IV included the test Number Series in the GIA, which was not a test in the GIA on the WJ III. Although not specifically a mathematics test, the task requires examinees to review a series of numbers where one number is missing and determine what number would complete the pattern. This test is very similar to the Number Matrices test included in the Math Problem Solving composite for the WJ IV, where examinees review a matrix of numbers and determine what number would correctly complete the matrix. This task similarity may have inflated the correlations between the GIA and the mathematics composites.

Consistent with previous investigations (e.g., McGrew and Wendling 2010), we also found several broad cognitive abilities, including G*c* and G*f* and to a lesser extent G*s* and G*wm*, significantly predicted mathematics skills relatively consistently across samples. In the integrated model, Math Calculation had large effects on Math Problem Solving, which was not surprising. This is important because the model implicitly assumes that basic calculation are important foundational skills for math problem solving skills, much like basic reading skills are important foundation skills needed for adequate reading comprehension (e.g., Hajovsky et al. 2014).

Although there remains considerable debate about the predictive validity of broad cognitive ability scores over and above *g* (see Breit et al. 2024), our results suggest that broad cognitive abilities are not inconsequential in how much variability they explain in mathematics. What is most important here, however, is that the relative importance of *g* and the broad cognitive abilities differ depending on an individual’s general cognitive ability level. When the variance accounted for in Math Calculation and Math Problem Solving by *g* and broad cognitive abilities were examined separately, we found substantial differences in the amount of variance explained by *g* and the broad cognitive abilities across the different ability groups. We observed that *g* explained more unique variance in mathematics for the low ability groups while broad cognitive abilities uniquely explained more variance in mathematics for those in the average and high ability groups. Interestingly, these results contrast with those by McGill (2015), who found that, for low ability individuals, broad cognitive abilities were *more* explanatory of math achievement. These discrepant findings may be attributable to the differences in the samples analyzed. The current study used elementary and secondary samples of the WJ III and WJ IV tests and McGill (2015) only used an aggregated WJ III normative sample (with ages ranging from 2 to 90 years old). It may also be related to how the groups were selected. McGill (2015) selected the groups using the GIA, a variable that was also used in the analysis. In the current study, an alternative GIA was used to select groups to help avoid the range restriction that can occur when groups are selected using variables that are included in the analysis. It should also be noted that the current results were observed only for the WJ III secondary and WJ IV elementary samples. Thus, future cognitive–achievement studies may need to consider the influence of age in addition to differences in tests across batteries.

### 4.2. Theoretical Implications

SLODR suggests that as general ability level increases, individuals become less dependent on their general capacity to navigate tasks, with broad cognitive abilities being therefore more differentiated (Blum and Holling 2017). Previous work on such effects has been expanding since 1989, when Detterman and Daniel found evidence supporting Spearman’s (1927) original testaments of a SLODR phenomenon (see Carrigan 2023). Since then, several others have found *g* to be less predictive of general ability as it increases, observing that its structure indeed becomes less cohesive (Kane et al. 2006; Reynolds et al. 2010).

Despite the evidence surrounding SLODR and intelligence, the literature is sparse and mixed as to how well SLODR applies to cognitive–achievement relations (Carrigan 2023). Among the few existing studies, Coyle et al. (2011) found that *g* better explained students’ grade point averages (GPAs) for those with high ability. Caemmerer and colleagues (2024) found that cognitive-achievement relations did not differ across different levels of general intelligence as measured by the WISC-V and WIAT-III, although there were notable differences between the current study and that by Caemmerer et al. (2024) regarding sample size and analytical approach. In contrast, Hajovsky et al. (2023) found that *g* was a relatively stronger predictor of reading skills for those with lower general ability and broad cognitive abilities were a relatively stronger predictor of reading for those with higher general ability. The findings from the current study align with these latter results, suggesting that as general cognitive ability increases, broad cognitive abilities become more predictive of academic skills. As SLODR predicts, for individuals whose broad cognitive abilities were highly intercorrelated (low ability groups), *g* explained more variance in mathematics skills. On the other hand, for those whose broad cognitive abilities were less intercorrelated (average and high ability groups), the abilities themselves explained more variance in mathematics skills.

Two common conceptualizations of SLODR effects on intelligence include sampling theory (Thurstone 1938) and the theory of minimal cognitive architecture (Anderson 1992). According to sampling theory, lower-ability individuals have fewer cognitive resources, limiting the complexity of their behaviors (e.g., test performance) and thus producing ability scores that are more intercorrelated (Carrigan 2023; Detterman and Daniel 1989; Thurstone 1938). Similarly, in the theory of minimal cognitive architecture, general processing (*g*) functions as a restrictor of specific cognitive abilities, such that more optimized general processing frees up specialized abilities (e.g., fluid reasoning) to become more efficient in each of their specific domains, and, as a result, shed their overlap with one another (Anderson 1992; Carrigan 2023). In consideration of our findings, broad cognitive abilities may have explained relatively more variance in mathematics skills for those with high ability levels because these individuals had more cognitive resources and less constraints on specialized mathematics skills, freeing up their use. Broad cognitive abilities were thus more differentiated and uniquely predictive of mathematics performance, while for low ability students whose broad cognitive abilities were more constrained by weaker general processing *g* remained a more robust predictor of mathematics skills.

The current findings may also be considered in light of mutualism, which asserts that reciprocal activity between academic and cognitive skills enhance each other’s growth (Peng and Kievit 2020). It has been theorized that students with higher ability levels are better equipped to generate mutualism among skills through their educational experiences (Peng and Kievit 2020). Such students may thus be more likely to pursue challenging tasks and further improve their academic knowledge while those with low abilities, who may find such tasks more demanding and resort to avoidance or withdrawal, reducing opportunities for building their academic knowledge (McGee et al. 2002; Zhang and Peng 2023). Students with high general intelligence are believed to learn more quickly and identify patterns across information more easily (Gottfredson 1997). For such individuals, broad cognitive abilities associated with general intelligence (e.g., fluid reasoning) may have a stronger association with academic skills (Caemmerer et al. 2024). On the other hand, students with lower general intelligence may recruit more processing-level skills to attend to, comprehend, encode, and recall new academic information (Henry and Winfield 2010). Related to mathematics skills, it has been proposed that mathematics learning first involves symbol association (e.g., the word “one” maps onto the symbol “1”) and progressively requires more complex, contextual, and “real world” problem solving skills (Decker and Roberts 2015). Our results suggest that for lower ability individuals general intelligence may maintain its influence on learning, while, for those with average or high general intelligence domain-specific processes play a gradually larger role. This could mean poorer academic trajectories for those who remain reliant on a general capacity given that mathematics content becomes relatively more complex and novel across grade levels compared to subjects like reading (Chu et al. 2016).

### 4.3. Practical Implications

In schools across the United States, it is common for students to be placed in intervention groups that target specific academic skills that require more intensive instruction (Zhang et al. 2023). Considering the high correlation between general mathematics skills and general intelligence (Zaboski et al. 2018), it is possible that many students who are in remedial mathematics intervention groups may be similar to those in the low general ability group in this study. If this is the case, it may be beneficial to focus on developing cognitive skills that were shown to be more highly related to mathematics for this group, such as fluid reasoning in the WJ III elementary sample. A focus on developing reasoning skills has been found to increase math performance in young children (e.g., Sterner et al. 2020), supporting this possibility.

Youth with more significant mathematics difficulties, such as developmental dyscalculia, mathematics difficulties, and low mathematics achievement, have been found to have lower scores on some broad cognitive abilities like working memory, in addition to their mathematics calculation and reasoning skills (Träff et al. 2020; Viesel-Nordmeyer et al. 2023). Gathering information regarding specific areas of weaknesses can help build a student’s comprehensive cognitive profile, predict their academic trajectory, and determine the need for special education services (Lewis and Fisher 2016). However, it remains poorly understood as to which broad cognitive abilities should be emphasized, as individuals with mathematics difficulties display a wide range of cognitive strengths and weaknesses (Peng et al. 2018). In turn, authors have called for studies to help differentiate which cognitive abilities matter for which individuals, particularly by examining their ability levels (i.e., Haberstroh and Schulte-Körne 2022). Our study goes some way towards this goal given that it builds on the few existing cognitive–achievement studies in the domain of mathematics and is the first to examine SLODR in mathematics with the WJ III and WJ IV specifically. In the realm of special education services, the process of identifying specific learning disabilities (SLD; e.g., dyscalculia) often involves interpreting the potential relations between a student’s cognitive and achievement test scores. The current findings add to the growing push for more nuanced models of identification, particularly those that account for a student’s ability level. For instance, if a student displays a high level of *g* on a WJ III or WJ IV battery (as indicated by the GIA), practitioners may need to place more interpretive weight on the broad cognitive ability scores when making decisions about disability status (Carrigan 2023). On the other hand, if the student has lower general ability scores, practitioners may consider placing more weight on the overall general intelligence score (Carrigan 2023).

### 4.4. Limitations and Future Research Directions

The findings reported from this study should be interpreted within the context of several important limitations. The first limitation concerns sampling issues. Data used in this research are from the WJ III and WJ IV normative standardization samples. Although these are large-scale datasets that were intended to be representative of the U.S. population, the findings generated from these data may not generalize to those with mathematics difficulties or those with a mathematics disability (e.g., dyscalculia). Future research should examine these findings with more heterogeneous samples and special populations, specifically those with demonstrated mathematics difficulties and/or are highly gifted in mathematics. Additionally, this research uses the same samples and methods as Hajovsky et al. (2023), only this study examines difference in cognitive–achievement relations across the general ability spectrum for mathematics, whereas the previous study examined these differences for reading. It is possible that the similar results found across these two studies are partially due to the use of the same samples and similar methodology. More research should be conducted with other popular and widely used comprehensive cognitive assessments to determine whether salient findings generalize across tests. Although key findings were replicated across two separate versions of the WJ using different samples, the strength of results would be increased with the analysis of additional tests. Cross-battery cognitive–achievement relations research, which is the simultaneous analysis of several intelligence and achievement test batteries (e.g., Caemmerer et al. 2023), is one future avenue to address this limitation.

The use of standardized, norm-referenced tests in this research has many advantages (e.g., co-normed tests, large nationally representative samples). It may be argued that the mathematics tests included on the WJ batteries are measures of mathematics “ability” rather than mathematics “achievement”. This is due to the lack of connection between what content is included on the WJ mathematics tests and whether that content is consistent with what students learn in the classroom. The WJ mathematics tests are purposefully broad in their content and are meant to sample a wide range mathematics concepts and skills. Tests that are designed to measure the acquisition of mathematics skills through classroom learning will be more focused and directly tied to what the students are exposed to in the classroom. Those tests may have different relations with cognitive abilities, and so these results may not generalize well to those types of assessments. Tests that are directly tied to mathematics instruction may have greater ecological validity, but this also introduces limitations because classroom instruction looks different across school districts and curricula used in those school districts. Although many states in the US have standards meant to guide instruction, the way in which those standards are taught can vary. Future research examining how these different types of mathematics tests are related to cognitive abilities would be helpful for better understanding whether there are substantive differences in those relations depending on how mathematics achievement is measured.

There are important measurement limitations of the study. The ability group selection procedure was based on pre-determined cut scores, which may not represent “low” and “high” ability well. Instead, the analysis of SLODR within the context of cognitive-math achievement relations may require more of a continuum when considering moderation by IQ level. Creating latent classes to represent different ability groups prior to examining cognitive–math achievement relations may help reduce Type II error issues specific to forming a priori groups based on cut scores (e.g., Reynolds et al. 2011). In addition, nearly all the analyses included observed variables rather than latent variables, which typically have a closer alignment with the construct of interest and remove measurement error to better estimate relations between constructs.

Finally, there are limitations given the assumptions of the models. Broadly speaking, assertions about the effects of one variable on another are dependent on the validity of the implied model. If the model is a reasonable approximation of reality, then the estimates derived from the model show the extent and magnitude of influence between variables. Our results are based on the analysis of effects within a theoretically defined model. The use of integrated models assumes the effects of cognitive abilities on mathematical problem solving are partially mediated via mathematical calculation skills (Villeneuve et al. 2019). There are two important assumptions being made here. First, mathematics is often modeled as a single latent construct (e.g., Taub et al. 2008), although there has been evidence that mathematics calculation and mathematical problem solving can be separated into different, albeit highly related, constructs (e.g., Thurber et al. 2002). We feel that the integrated model that separates mathematical calculation and problem solving is warranted because foundational calculation skills are necessary precursors to more complex problem-solving skills. Second, because we used cross-sectional data, it is assumed mediation occurs instantaneously (Cole and Maxwell 2003; Preacher 2015), which is unlikely and may not represent a true mediation effect when considering how variables are related to each other. Although the scope and focus of our study was on moderation, well-designed longitudinal studies and statistical models should be used when addressing substantive questions related to mediation.

Examining cognitive–achievement relations in different ability groups provides some interesting insights into how academic skills develop. The current study found that general intelligence explained relatively more variance in mathematics skills for those with lower ability, whereas broad cognitive abilities explained relatively more variance in mathematics skills for those with average or higher ability in integrated models of cognitive abilities and mathematics skills. These findings are consistent with theoretical predictions and add to a limited body of literature that suggests cognitive–achievement relations vary by general ability level. Empirically, our findings show that as general ability level increases, broad cognitive abilities become more predictive of mathematics skills. Practically, the actual application of these results to clinical assessment requires further scrutiny, but our findings may suggest an eventual reworking of how cognitive–achievement relations are interpreted in light of general cognitive ability. However, given our study results that take overall ability level into account when examining cognitive–achievement relations, further research is warranted.

## Figures and Tables

**Table 1:** Description of broad CHC cognitive abilities and tests measured on the WJ III and WJ IV.

Broad Ability	Description	Tests
Comprehension Knowledge (G*c*)	The ability to apply knowledge derived from life experiences and education.	WJ III: Verbal Comprehension and General Information WJ IV: Oral Vocabulary and General Information
Fluid Reasoning (G*f*)	The ability to reason, form concepts, and solve problems using new information or procedures.	WJ III: Concept Formation and Analysis Synthesis WJ IV: Number Series and Concept Formation
Visual Processing (G*v*)	The ability to analyze, synthesize, and think using visual patterns.	WJ III: Spatial Relations and Picture Recognition WJ IV: Visualization and Picture Recognition
Auditory Processing (G*a*)	The ability to synthesize and discriminate auditory stimuli and employ auditory information during tasks.	WJ III: Sound Blending and Auditory Attention WJ IV: Phonological Processing and Nonword Repetition
Long-term Retrieval (G*lr*)	The ability to store information from short-term memory and draw on it later in the process of thinking	WJ III: Visual-Auditory Learning and Retrieval Fluency WJ IV: Story Recall and Visual-Auditory Learning
Short-term Working Memory (G*wm*)	The ability to capture and retain information in and use or manipulate it to complete a goal.	WJ III: Numbers Reversed and Memory for Words WJ IV: Verbal Attention and Numbers Reversed
Cognitive Processing Speed (G*s*)	The ability to quickly perform simple and complex tasks, especially when having to sustain controlled attention and concentration.	WJ III: Visual Matching and Decision Speed WJ IV: Letter Pattern Matching and Pair Cancellation

**Table 2:** Descriptive statistics for the WJ III.

	Low-Ability Group				Average-Ability Group				High-Ability Group			
	*N*	*M* (*SD*)	Skew	Kurt	*N*	*M* (*SD*)	Skew	Kurt	*N*	*M* (*SD*)	Skew	Kurt
WJ III Elementary												
G*c*	302	83.03 (11.70)	−0.64	0.48	658	101.07 (9.03)	0.07	−0.04	362	114.07 (8.57)	−0.11	0.29
G*lr*	306	87.67 (10.61)	−0.28	0.47	659	101.30 (10.29)	0.30	0.63	353	112.93 (10.70)	0.33	0.10
G*v*	277	93.28 (14.18)	−0.17	−0.34	656	99.63 (13.74)	−0.38	0.71	378	107.27 (14.13)	−0.08	1.03
G*a*	303	89.86 (13.98)	−0.27	0.18	614	101.57 (13.07)	−0.01	0.18	337	111.96 (13.27)	0.08	0.06
G*f*	369	83.67 (13.21)	−0.64	0.18	832	100.11 (10.62)	−0.35	0.67	454	114.30 (10.84)	0.24	0.33
G*s*	343	90.62 (13.87)	−0.28	0.46	701	100.98 (12.30)	−0.14	0.10	364	107.30 (14.01)	0.21	0.12
G*wm*	400	85.74 (13.49)	−0.13	0.87	883	100.75 (12.61)	0.01	0.28	463	111.63 (13.67)	0.50	1.33
MC	390	89.57 (15.85)	−0.45	0.55	866	100.11 (13.46)	−0.11	0.47	421	108.54 (14.05)	0.43	0.93
MPS	306	85.40 (11.80)	−0.41	0.11	681	100.71 (10.66)	0.15	−0.10	355	113.43 (12.34)	0.29	0.69
GIA	249	81.57 (8.71)	−0.80	0.99	614	99.38 (6.98)	0.07	−0.14	363	117.20 (9.53)	0.87	1.51
WJ III Secondary												
G*c*	326	83.26 (11.24)	−0.65	0.88	686	100.98 (9.13)	−0.40	2.14	391	115.65 (9.67)	0.48	0.42
G*lr*	301	85.57 (11.96)	−0.17	0.23	625	98.91 (11.01)	0.47	1.31	347	112.98 (11.79)	0.62	0.30
G*v*	310	90.10 (13.00)	−0.15	−0.22	623	100.01 (12.19)	−0.21	0.40	358	109.39 (13.45)	−0.02	−0.02
G*a*	271	86.48 (12.37)	0.04	0.38	505	99.47 (12.20)	0.17	0.55	285	109.27 (14.21)	0.55	0.43
G*f*	331	83.15 (11.79)	−0.33	0.27	704	100.24 (10.27)	−0.17	0.27	386	115.28 (9.02)	0.01	−0.05
G*s*	419	90.64 (14.92)	−0.12	0.87	849	100.12 (13.68)	−0.08	0.30	467	108.05 (14.41)	0.14	−0.05
G*wm*	426	87.62 (13.03)	−0.15	0.12	866	101.53 (11.52)	−0.02	0.37	470	112.64 (12.03)	0.11	0.15
MC	471	88.42 (15.27)	−0.35	0.39	1031	100.75 (12.97)	−0.03	0.11	536	110.75 (12.64)	0.20	0.26
MPS	323	82.48 (12.38)	−0.61	0.90	691	100.49 (10.20)	0.17	−0.13	378	115.78 (12.20)	0.15	−0.03
GIA	292	82.70 (9.52)	−1.13	1.50	604	101.79 (6.94)	0.07	−0.51	341	120.82 (9.08)	0.80	0.60

*Note*. G*c* = Comprehension–Knowledge, G*lr* = Long-Term Retrieval, G*v* = Visual Processing, G*a* = Auditory Processing, G*f* = Fluid Reasoning, G*s* = Processing Speed, G*wm* = Short-Term Working Memory, MC = Math Calculation, MPS = Math Problem Solving, GIA = General Intellectual Ability, Skew = skewness, Kurt = kurtosis.

**Table 3:** Descriptive statistics for the WJ IV.

	Low-Ability Group				Average-Ability Group				High-Ability Group			
	*N*	*M* (*SD*)	Skew	Kurt	*N*	*M* (*SD*)	Skew	Kurt	*N*	*M* (*SD*)	Skew	Kurt
WJ IV Elementary												
G*c*	400	86.27 (13.22)	0.07	−0.26	801	100.14 (12.09)	−0.12	0.69	412	111.83 (12.38)	0.11	−0.03
G*lr*	401	86.13 (12.67)	−0.13	0.04	801	100.69 (12.43)	−0.21	0.20	412	112.55 (12.36)	0.13	0.20
G*v*	401	87.91 (14.01)	−0.30	0.39	801	101.13 (12.87)	0.11	0.69	412	111.95 (13.97)	0.23	0.20
G*a*	401	84.43 (12.26)	−0.07	0.88	801	99.85 (11.94)	0.13	0.17	412	114.03 (11.29)	0.07	0.19
G*f*	401	83.95 (11.56)	−0.40	0.70	801	100.39 (9.93)	0.07	−0.02	412	115.16 (11.57)	0.25	0.10
G*s*	399	89.15 (14.33)	0.16	0.69	799	100.53 (13.05)	−0.01	0.42	412	110.04 (13.34)	0.26	0.36
G*wm*	400	86.03 (12.50)	−0.38	0.79	801	100.62 (11.03)	−0.09	0.20	412	114.27 (11.69)	0.00	0.78
MC	395	86.59 (14.15)	−0.34	0.88	801	100.54 (11.47)	0.11	0.36	412	113.08 (12.71)	0.62	0.67
MPS	400	85.30 (12.14)	−0.36	0.59	801	100.64 (10.37)	0.21	−0.14	412	115.14 (11.10)	0.27	1.17
GIA	401	80.14 (9.57)	−1.06	1.49	801	100.39 (6.45)	0.13	−0.34	412	118.37 (7.62)	1.23	2.48
WJ IV Secondary												
G*c*	466	86.43 (12.77)	−0.01	0.48	997	99.72 (11.66)	0.11	0.19	492	115.14 (12.71)	0.42	0.18
G*lr*	466	87.11 (13.97)	−0.12	0.16	997	100.35 (12.34)	0.17	0.06	492	112.35 (13.18)	0.15	−0.13
G*v*	466	88.71 (14.34)	−0.05	0.51	997	101.07 (13.03)	0.17	0.34	492	111.13 (14.02)	0.31	0.07
G*a*	466	84.55 (11.85)	−0.09	0.18	997	100.47 (11.46)	0.18	−0.26	492	114.14 (12.62)	0.20	−0.01
G*f*	466	82.82 (11.68)	−0.33	0.21	997	99.81 (11.32)	0.00	0.28	492	114.04 (10.91)	0.08	−0.16
G*s*	466	87.59 (14.95)	−0.40	0.66	997	99.60 (12.81)	−0.10	0.13	492	110.45 (12.69)	−0.10	−0.22
G*wm*	466	85.97 (11.31)	−0.09	0.24	997	100.54 (11.99)	0.04	0.00	492	115.16 (11.73)	0.22	−0.06
MC	466	85.38 (14.41)	−0.65	1.76	997	100.60 (12.51)	0.01	0.23	492	112.96 (13.01)	0.14	0.34
MPS	466	84.45 (12.12)	−0.39	0.51	997	100.72 (11.01)	−0.13	−0.08	492	114.93 (11.22)	0.26	0.02
GIA	466	79.28 (9.57)	−1.24	2.22	997	99.52 (6.90)	0.02	−0.54	492	118.21 (7.66)	0.67	0.27

*Note*. G*c* = Comprehension–Knowledge, G*lr* = Long-Term Retrieval, G*v* = Visual Processing, G*a* = Auditory Processing, G*f* = Fluid Reasoning, G*s* = Processing Speed, G*wm* = Short-Term Working Memory, MC = Math Calculation, MPS = Math Problem Solving, GIA = General Intellectual Ability, Skew = skewness, Kurt = kurtosis.

**Table 4:** Coefficients for model 1.

	Low-Ability Group		Average-Ability Group		High-Ability Group		Pairwise Comparisons
	*b* (SE)	β (SE)	*b* (SE)	β (SE)	*b* (SE)	β (SE)	
WJ III Elementary							
GIA→MC	**0.87 (0.10)**	**0.48 (0.05)**	**0.51 (0.08)**	**0.27 (0.04)**	**0.42 (0.09)**	**0.28 (0.06)**	L > A, L > H, A = H
MC→MP	**0.37 (0.04)**	**0.49 (0.05)**	**0.26 (0.03)**	**0.33 (0.04)**	**0.37 (0.05)**	**0.42 (0.05)**	L > A, L = H, A < H
GIA→MP	**0.31 (0.08)**	**0.23 (0.06)**	**0.28 (0.06)**	**0.18 (0.04)**	**0.22 (0.07)**	**0.17 (0.06)**	L = A, L = H, A = H
MC *R*^2^	0.23 (0.05)		0.07 (0.02)		0.08 (0.03)		L = A, L = H, A = H
MP *R*^2^	0.41 (0.05)		0.17 (0.03)		0.24 (0.04)		L = A, L = H, A = H
WJ III Secondary							
GIA→MC	**0.68 (0.08)**	**0.43 (0.05)**	**0.43 (0.07)**	**0.23 (0.04)**	**0.41 (0.07)**	**0.30 (0.05)**	L > A, L > H, A = H
MC→MP	**0.40 (0.04)**	**0.49 (0.04)**	**0.33 (0.03)**	**0.42 (0.03)**	**0.50 (0.04)**	**0.52 (0.04)**	L = A, L = H, A < H
GIA→MP	**0.47 (0.06)**	**0.36 (0.05)**	**0.28 (0.05)**	**0.19 (0.04)**	**0.28 (0.06)**	**0.21 (0.05)**	L > A, L > H, A = H
MC *R*^2^	0.19 (0.04)		0.05 (0.02)		0.09 (0.03)		L = A, L = H, A = H
MP *R*^2^	0.52 (0.04)		0.25 (0.03)		0.38 (0.04)		L = A, L = H, A = H
WJ IV Elementary							
GIA→MC	**0.95 (0.06)**	**0.64 (0.03)**	**0.99 (0.05)**	**0.56 (0.02)**	**0.85 (0.07)**	**0.51 (0.04)**	L = A, L = H, A = H
MC→MP	**0.37 (0.04)**	**0.43 (0.05)**	**0.34 (0.03)**	**0.38 (0.04)**	**0.34 (0.04)**	**0.39 (0.05)**	L = A, L = H, A = H
GIA→MP	**0.40 (0.06)**	**0.32 (0.05)**	**0.28 (0.06)**	**0.17 (0.04)**	**0.37 (0.07)**	**0.25 (0.05)**	L = A, L = H, A = H
MC *R*^2^	0.40 (0.04)		0.31 (0.03)		0.26 (0.04)		L = A, L = H, A = H
MP *R*^2^	0.46 (0.04)		0.25 (0.03)		0.32 (0.04)		L = A, L = H, A = H
WJ IV Secondary							
GIA→MC	**0.99 (0.05)**	**0.66 (0.03)**	**0.92 (0.05)**	**0.51 (0.02)**	**0.88 (0.07)**	**0.52 (0.03)**	L = A, L = H, A = H
MC→MP	**0.33 (0.04)**	**0.39 (0.04)**	**0.34 (0.03)**	**0.39 (0.03)**	**0.35 (0.04)**	**0.40 (0.04)**	L = A, L = H, A = H
GIA→MP	**0.44 (0.06)**	**0.35 (0.04)**	**0.45 (0.05)**	**0.28 (0.03)**	**0.38 (0.06)**	**0.26 (0.04)**	L = A, L = H, A = H
MC *R*^2^	0.43 (0.04)		0.26 (0.02)		0.27 (0.03)		L = A, L = H, A = H
MP *R*^2^	0.45 (0.03)		0.34 (0.02)		0.34 (0.04)		L = A, L = H, A = H

*Note*. MC = Math Calculation, MPS = Math Problem Solving, GIA = General Intellectual Ability, L = low ability group, A = average ability group, H = high ability group. Bold values are statistically significant (*p* < .05).

**Table 5:** Coefficients for model 2 with the WJ III.

	Low-Ability Group		Average-Ability Group		High-Ability Group		Pairwise Comparisons
	*b* (SE)	β (SE)	*b* (SE)	β (SE)	*b* (SE)	β (SE)	
WJ III Elementary							
MC→MPS	**0.37 (0.04)**	**0.49 (0.05)**	**0.23 (0.03)**	**0.30 (0.04)**	**0.37 (0.05)**	**0.42 (0.05)**	L > A, L = H, A < H
G*c*→MPS	**0.23 (0.05)**	**0.22 (0.05)**	**0.22 (0.04)**	**0.19 (0.04)**	*0.07 (0.07)*	*0.05 (0.05)*	L = A, L = H, A = H
G*lr*→MPS	*0.06 (0.06)*	*0.06 (0.05)*	*0.04 (0.04)*	*0.04 (0.04)*	*0.05 (0.06)*	*0.04 (0.05)*	L = A, L = H, A = H
G*v*→MPS	*0.04 (0.04)*	*0.04 (0.05)*	*0.03 (0.03)*	*0.04 (0.04)*	**0.09 (0.05)**	**0.10 (0.05)**	L = A, L = H, A = H
G*a*→MPS	*−0.09 (0.06)*	*−0.11 (0.07)*	*−0.01 (0.04)*	*−0.01 (0.05)*	*−0.02 (0.06)*	*−0.02 (0.06)*	L = A, L = H, A = H
G*f*→MPS	**0.10 (0.04)**	**0.11 (0.05)**	**0.12 (0.04)**	**0.12 (0.04)**	**0.16 (0.05)**	**0.14 (0.05)**	L = A, L = H, A = H
G*s*→MPS	*−0.01 (0.05)*	*−0.01 (0.05)*	**0.10 (0.04)**	**0.11 (0.04)**	*−0.02 (0.05)*	*−0.02 (0.05)*	L = A, L = H, A = H
G*wm*→MPS	*0.04 (0.04)*	*0.04 (0.05)*	**0.06 (0.03)**	**0.07 (0.04)**	**0.13 (0.04)**	**0.14 (0.05)**	L = A, L = H, A = H
G*c*→MC	**0.19 (0.08)**	**0.14 (0.06)**	*0.02 (0.06)*	*0.02 (0.04)*	*0.07 (0.08)*	*0.04 (0.05)*	L = A, L = H, A = H
G*lr*→MC	*0.08 (0.10)*	*0.06 (0.07)*	*0.11 (0.06)*	*0.08 (0.04)*	*0.06 (0.08)*	*0.05 (0.06)*	L = A, L = H, A = H
G*v*→MC	*−0.06 (0.06)*	*−0.05 (0.06)*	*−0.04 (0.05)*	*−0.04 (0.05)*	*−0.08 (0.06)*	*−0.08 (0.06)*	L = A, L = H, A = H
G*a*→MC	*−0.06 (0.07)*	*−0.05 (0.06)*	*0.01 (0.05)*	*0.01 (0.05)*	*−0.01 (0.07)*	*−0.01 (0.07)*	L = A, L = H, A = H
G*f*→MC	**0.15 (0.07)**	**0.13 (0.06)**	**0.17 (0.05)**	**0.14 (0.04)**	*0.11 (0.07)*	*0.08 (0.05)*	L = A, L = H, A = H
G*s*→MC	**0.45 (0.06)**	**0.38 (0.05)**	**0.37 (0.04)**	**0.34 (0.03)**	**0.35 (0.05)**	**0.35 (0.05)**	L = A, L = H, A = H
G*wm*→MC	**0.19 (0.06)**	**0.17 (0.05)**	**0.08 (0.04)**	**0.08 (0.04)**	**0.22 (0.05)**	**0.22 (0.05)**	L = A, L = H, A < H
*g*→MC	**5.11 (0.69)**	**0.33 (0.04)**	**3.73 (0.68)**	**0.10 (0.02)**	**3.91 (0.88)**	**0.13 (0.03)**	
*g*→MP	**4.33 (0.55)**	**0.36 (0.04)**	**4.15 (0.56)**	**0.14 (0.03)**	**4.11 (0.79)**	**0.16 (0.04)**	
*R*^2^ MC	0.30 (0.04)		0.16 (0.03)		0.20 (0.04)		
*R*^2^ MP	0.44 (0.05)		0.21 (0.03)		0.27 (0.04)		
WJ III Secondary							
MC→MPS	**0.39 (0.03)**	**0.47 (0.04)**	**0.34 (0.03)**	**0.44 (0.03)**	**0.51 (0.04)**	**0.53 (0.04)**	L = A, L < H, A < H
G*c*→MPS	**0.38 (0.04)**	**0.36 (0.04)**	**0.28 (0.04)**	**0.25 (0.03)**	**0.25 (0.05)**	**0.20 (0.04)**	L = A, L = H, A = H
G*lr*→MPS	**−0.14 (0.04)**	**−0.13 (0.04)**	*0.03 (0.03)*	*0.03 (0.03)*	*−0.02 (0.04)*	*−0.02 (0.04)*	L < A, L = H, A = H
G*v*→MPS	*0.06 (0.04)*	*0.06 (0.04)*	*−0.02 (0.03)*	*−0.02 (0.03)*	*0.03 (0.04)*	*0.03 (0.04)*	L = A, L = H, A = H
G*a*→MPS	*−0.01 (0.05)*	*−0.01 (0.05)*	*−0.06 (0.04)*	*−0.07 (0.05)*	*−0.06 (0.04)*	*−0.06 (0.05)*	L = A, L = H, A = H
G*f*→MPS	**0.24 (0.04)**	**0.22 (0.04)**	**0.23 (0.03)**	**0.23 (0.03)**	**0.29 (0.06)**	**0.21 (0.04)**	L = A, L = H, A = H
G*s*→MPS	*0.03 (0.03)*	*0.04 (0.04)*	**−0.08 (0.03)**	**−0.11 (0.03)**	**−0.08 (0.03)**	**−0.09 (0.04)**	L > A, L > H, A = H
G*wm*→MPS	**0.12 (0.04)**	**0.12 (0.04)**	*0.04 (0.03)*	*0.05 (0.03)*	*0.03 (0.04)*	*0.03 (0.04)*	L = A, L = H, A = H
G*c*→MC	**0.15 (0.07)**	**0.12 (0.06)**	*0.08 (0.05)*	*0.06 (0.04)*	**0.15 (0.06)**	**0.12 (0.05)**	L = A, L = H, A = H
G*lr*→MC	*0.04 (0.07)*	*0.03 (0.06)*	*−0.03 (0.05)*	*−0.03 (0.04)*	*−0.03 (0.05)*	*−0.03 (0.05)*	L = A, L = H, A = H
G*v*→MC	*−0.04 (0.06)*	*−0.04 (0.05)*	*0.00 (0.04)*	*0.00 (0.04)*	*0.05 (0.05)*	*0.06 (0.05)*	L = A, L = H, A = H
G*a*→MC	**−0.17 (0.08)**	**−0.14 (0.06)**	**−0.17 (0.04)**	**−0.16 (0.04)**	*−0.03 (0.05)*	*−0.03 (0.06)*	L = A, L = H, A < H
G*f*→MC	**0.20 (0.07)**	**0.16 (0.06)**	**0.18 (0.05)**	**0.14 (0.04)**	**0.27 (0.07)**	**0.19 (0.05)**	L = A, L = H, A = H
G*s*→MC	**0.30 (0.05)**	**0.29 (0.05)**	**0.29 (0.03)**	**0.31 (0.03)**	**0.24 (0.04)**	**0.27 (0.04)**	L = A, L = H, A = H
G*wm*→MC	**0.22 (0.06)**	**0.19 (0.05)**	**0.11 (0.04)**	**0.09 (0.03)**	*0.03 (0.05)*	*0.03 (0.04)*	L = A, L > H, A = H
*g*→MC	**3.97 (0.64)**	**0.26 (0.04)**	**2.48 (0.59)**	**0.08 (0.02)**	**3.92 (0.69)**	**0.18 (0.03)**	
*g*→MPS	**5.91 (0.56)**	**0.47 (0.04)**	**4.09 (0.51)**	**0.16 (0.03)**	**5.28 (0.70)**	**0.25 (0.03)**	
*R*^2^ MC	0.22 (0.04)		0.15 (0.03)		0.15 (0.03)		
*R*^2^ MPS	0.64 (0.03)		0.36 (0.03)		0.46 (0.04)		

*Note*. G*c* = Comprehension–Knowledge, G*lr* = Long-Term Retrieval, G*v* = Visual Processing, G*a* = Auditory Processing, G*f* = Fluid Reasoning, G*s* = Processing Speed, G*wm* = Short-Term Working Memory, MC = Math Calculation, MPS = Math Problem Solving, *g* = general intelligence, L = low ability group, A = average ability group, H = high ability group. Bold values are statistically significant (*p* < .05). Italicized values are not statistically significant (*p* > .05).

**Table 6:** Coefficients for model 2 with the WJ IV.

	Low-Ability Group		Average-Ability Group		High-Ability Group		Pairwise Comparisons
	*b* (SE)	β (SE)	*b* (SE)	β (SE)	*b* (SE)	β (SE)	
WJ IV Elementary							
MC→MPS	**0.36 (0.04)**	**0.42 (0.05)**	**0.32 (0.03)**	**0.35 (0.03)**	**0.33 (0.04)**	**0.38 (0.04)**	L = A, L = H, A = H
G*c*→MPS	**0.11 (0.04)**	**0.12 (0.04)**	**0.08 (0.02)**	**0.10 (0.03)**	**0.15 (0.04)**	**0.17 (0.04)**	L = A, L = H, A = H
G*lr*→MPS	*0.01 (0.04)*	*0.01 (0.04)*	*−0.01 (0.02)*	*−0.01 (0.03)*	**0.07 (0.04)**	**0.08 (0.04)**	L = A, L = H, A = H
G*v*→MPS	*0.05 (0.03)*	*0.06 (0.04)*	**0.05 (0.02)**	**0.06 (0.03)**	*−0.01 (0.03)*	*−0.01 (0.04)*	L = A, L = H, A = H
G*a*→MPS	*0.03 (0.04)*	*0.03 (0.04)*	*−0.03 (0.02)*	*−0.03 (0.03)*	*−0.02 (0.04)*	*−0.02 (0.04)*	L = A, L = H, A = H
G*f*→MPS	**0.36 (0.04)**	**0.35 (0.04)**	**0.42 (0.03)**	**0.41 (0.03)**	**0.37 (0.04)**	**0.38 (0.04)**	L = A, L = H, A = H
G*s*→MPS	**−0.07 (0.03)**	**−0.08 (0.04)**	**−0.09 (0.03)**	**−0.11 (0.03)**	*−0.02 (0.04)*	*−0.03 (0.04)*	L = A, L = H, A = H
G*wm*→MPS	*0.07 (0.04)*	*0.07 (0.04)*	**0.05 (0.03)**	**0.06 (0.03)**	*−0.02 (0.04)*	*−0.02 (0.04)*	L = A, L = H, A = H
G*c*→MC	**0.22 (0.04)**	**0.20 (0.04)**	**0.16 (0.03)**	**0.17 (0.03)**	**0.18 (0.04)**	**0.17 (0.04)**	L = A, L = H, A = H
G*lr*→MC	*0.00 (0.05)*	*0.00 (0.04)*	**−0.08 (0.03)**	**−0.09 (0.03)**	*−0.01 (0.04)*	*−0.01 (0.04)*	L = A, L = H, A = H
G*v*→MC	*−0.06 (0.04)*	*−0.06 (0.04)*	*0.02 (0.03)*	*0.03 (0.03)*	*0.01 (0.04)*	*0.01 (0.04)*	L = A, L = H, A = H
G*a*→MC	**−0.17 (0.05)**	**−0.15 (0.04)**	**−0.07 (0.03)**	**−0.07 (0.03)**	**−0.15 (0.05)**	**−0.13 (0.04)**	L < A, L = H, A = H
G*f*→MC	**0.55 (0.05)**	**0.46 (0.04)**	**0.41 (0.03)**	**0.36 (0.03)**	**0.31 (0.05)**	**0.29 (0.04)**	L > A, L > H, A = H
G*s*→MC	**0.35 (0.04)**	**0.35 (0.04)**	**0.38 (0.03)**	**0.43 (0.03)**	**0.36 (0.04)**	**0.38 (0.04)**	L = A, L = H, A = H
G*wm*→MC	**0.12 (0.05)**	**0.11 (0.04)**	**0.09 (0.03)**	**0.09 (0.03)**	*0.08 (0.05)*	*0.07 (0.04)*	L = A, L = H, A = H
*g*→MC	**5.49 (0.54)**	**0.38 (0.03)**	**4.85 (0.45)**	**0.23 (0.02)**	**3.97 (0.56)**	**0.22 (0.03)**	
*g*→MP	**5.46 (0.48)**	**0.44 (0.03)**	**4.61 (0.42)**	**0.24 (0.02)**	**4.45 (0.50)**	**0.28 (0.03)**	
*R*^2^ MC	0.47 (0.04)		0.38 (0.03)		0.30 (0.04)		
*R*^2^ MP	0.55 (0.03)		0.42 (0.03)		0.45 (0.04)		
WJ IV Secondary							
MC→MPS	**0.36 (0.03)**	**0.43 (0.04)**	**0.30 (0.03)**	**0.34 (0.03)**	**0.34 (0.04)**	**0.40 (0.04)**	L = A, L = H, A = H
G*c*→MPS	**0.11 (0.03)**	**0.12 (0.03)**	**0.16 (0.02)**	**0.17 (0.02)**	**0.15 (0.03)**	**0.17 (0.04)**	L = A, L = H, A = H
G*lr*→MPS	*−0.02 (0.03)*	*−0.02 (0.03)*	**−0.05 (0.02)**	**−0.06 (0.02)**	*0.02 (0.03)*	*0.02 (0.04)*	L = A, L = H, A = H
G*v*→MPS	*0.03 (0.03)*	*0.03 (0.03)*	*0.03 (0.02)*	*0.04 (0.02)*	*−0.01 (0.03)*	*−0.01 (0.04)*	L = A, L = H, A = H
G*a*→MPS	*0.03 (0.03)*	*0.03 (0.03)*	*0.01 (0.02)*	*0.01 (0.02)*	*0.02 (0.03)*	*0.02 (0.04)*	L = A, L = H, A = H
G*f*→MPS	**0.43 (0.04)**	**0.40 (0.04)**	**0.41 (0.03)**	**0.42 (0.02)**	**0.32 (0.04)**	**0.31 (0.04)**	L = A, L > H, A = H
G*s*→MPS	**−0.07 (0.03)**	**−0.09 (0.04)**	*−0.03 (0.02)*	*−0.03 (0.03)*	*−0.04 (0.03)*	*−0.04 (0.04)*	L = A, L = H, A = H
G*wm*→MPS	*0.05 (0.04)*	*0.04 (0.03)*	**0.04 (0.02)**	**0.05 (0.02)**	*0.05 (0.04)*	*0.06 (0.04)*	L = A, L = H, A = H
G*c*→MC	**0.27 (0.04)**	**0.24 (0.04)**	**0.16 (0.03)**	**0.15 (0.03)**	**0.12 (0.04)**	**0.12 (0.04)**	L > A, L > H, A = H
G*lr*→MC	*−0.02 (0.04)*	*−0.02 (0.04)*	**−0.09 (0.03)**	**−0.09 (0.03)**	*−0.03 (0.04)*	*−0.03 (0.04)*	L = A, L = H, A = H
G*v*→MC	*0.05 (0.04)*	*0.05 (0.04)*	*0.03 (0.03)*	*0.03 (0.03)*	*0.05 (0.04)*	*0.05 (0.04)*	L = A, L = H, A = H
G*a*→MC	*−0.07 (0.05)*	*−0.05 (0.04)*	**−0.11 (0.03)**	**−0.10 (0.03)**	**−0.12 (0.04)**	**−0.12 (0.04)**	L = A, L = H, A = H
G*f*→MC	**0.43 (0.05)**	**0.34 (0.04)**	**0.41 (0.03)**	**0.37 (0.03)**	**0.43 (0.05)**	**0.37 (0.04)**	L = A, L = H, A = H
G*s*→MC	**0.38 (0.04)**	**0.39 (0.03)**	**0.33 (0.03)**	**0.34 (0.03)**	**0.32 (0.04)**	**0.31 (0.04)**	L = A, L = H, A = H
G*wm*→MC	*0.03 (0.05)*	*0.02 (0.04)*	**0.12 (0.03)**	**0.12 (0.03)**	**0.10 (0.05)**	**0.09 (0.04)**	L = A, L = H, A = H
*g*→MC	**5.46 (0.51)**	**0.38 (0.03)**	**4.36 (0.41)**	**0.19 (0.02)**	**4.41 (0.51)**	**0.26 (0.03)**	
*g*→MPS	**5.19 (0.44)**	**0.42 (0.03)**	**4.59 (0.38)**	**0.22 (0.02)**	**4.48 (0.45)**	**0.30 (0.03)**	
*R*^2^ MC	0.42 (0.03)		0.33 (0.02)		0.30 (0.03)		
*R*^2^ MPS	0.56 (0.03)		0.46 (0.02)		0.43 (0.03)		

*Note*. G*c* = Comprehension–Knowledge, G*lr* = Long-Term Retrieval, G*v* = Visual Processing, G*a* = Auditory Processing, G*f* = Fluid Reasoning, G*s* = Processing Speed, G*wm* = Short-Term Working Memory, MC = Math Calculation, MPS = Math Problem Solving, *g* = general intelligence, L = low ability group, A = average ability group, H = high ability group. Bold values are statistically significant (*p* < .05). Italicized values are not statistically significant (*p* > .05).

**Table 7:** Relative variance accounted for in mathematics by *g* and broad cognitive abilities for the WJ III.

	*R* ^2^	Indirect Effect of *g* (Effect^2^)	Total Effects of Broad Abilities (Total Effect^2^)	Proportion of *R*^2^ Attributable to *g*	Proportion of *R*^2^ Attributable to Broad Abilities
WJ III Elementary					
Low-Ability Group					
Math Calculation	0.30	0.33 (0.11)	0.44 (0.19)	0.36	0.64
Math Problem Solving	0.44	0.36 (0.13)	0.56 (0.31)	0.29	0.71
Average-Ability Group					
Math Calculation	0.16	0.10 (0.01)	0.39 (0.15)	0.06	0.94
Math Problem Solving	0.21	0.14 (0.02)	0.44 (0.19)	0.09	0.91
High-Ability Group					
Math Calculation	0.20	0.13 (0.02)	0.43 (0.18)	0.08	0.92
Math Problem Solving	0.27	0.16 (0.03)	0.49 (0.24)	0.09	0.91
WJ III Secondary					
Low-Ability Group					
Math Calculation	0.22	0.26 (0.07)	0.39 (0.15)	0.31	0.69
Math Problem Solving	0.64	0.47 (0.22)	0.65 (0.42)	0.35	0.65
Average-Ability Group					
Math Calculation	0.15	0.08 (0.01)	0.38 (0.14)	0.04	0.96
Math Problem Solving	0.36	0.16 (0.03)	0.58 (0.33)	0.07	0.93
High-Ability Group					
Math Calculation	0.15	0.18 (0.03)	0.34 (0.12)	0.22	0.78
Math Problem Solving	0.46	0.25 (0.06)	0.63 (0.40)	0.14	0.86

**Table 8:** Relative variance accounted for in mathematics by *g* and broad cognitive abilities for the WJ IV.

	*R* ^2^	Indirect Effect of *g* (Effect^2^)	Total Effects of Broad Abilities (Total Effect^2^)	Proportion of *R*^2^ Attributable to *g*	Proportion of *R*^2^ Attributable to Broad Abilities
WJ IV Elementary					
Low-Ability Group					
Math Calculation	0.47	0.38 (0.14)	0.57 (0.33)	0.31	0.69
Math Problem Solving	0.55	0.44 (0.19)	0.60 (0.36)	0.35	0.65
Average-Ability Group					
Math Calculation	0.38	0.23 (0.05)	0.57 (0.33)	0.14	0.86
Math Problem Solving	0.42	0.24 (0.06)	0.60 (0.36)	0.14	0.86
High-Ability Group					
Math Calculation	0.30	0.22 (0.05)	0.50 (0.25)	0.16	0.84
Math Problem Solving	0.45	0.28 (0.08)	0.61 (0.37)	0.17	0.83
WJ IV Secondary					
Low-Ability Group					
Math Calculation	0.42	0.38 (0.14)	0.52 (0.28)	0.34	0.66
Math Problem Solving	0.56	0.42 (0.18)	0.62 (0.38)	0.32	0.69
Average-Ability Group					
Math Calculation	0.33	0.19 (0.04)	0.54 (0.29)	0.11	0.89
Math Problem Solving	0.46	0.22 (0.05)	0.64 (0.41)	0.11	0.89
High-Ability Group					
Math Calculation	0.30	0.26 (0.07)	0.48 (0.23)	0.23	0.77
Math Problem Solving	0.43	0.30 (0.09)	0.58 (0.34)	0.21	0.79

## Data Availability

The data are not publicly available and were used with permission from the Woodcock Institute for the Advancement of Neurocognitive Research and Applied Practice.

## References

[B1-jintelligence-13-00065] Anderson Mitchell (1992). Intelligence and Development: A Cognitive Theory.

[B2-jintelligence-13-00065] Blum Diego, Holling Heinz (2017). Spearman’s law of diminishing returns. A meta-analysis. Intelligence.

[B3-jintelligence-13-00065] Breit Moritz, Preckel Francis (2020). Incremental validity of specific cognitive abilities beyond general intelligence for the explanation of students’ school achievement. Gifted and Talented International.

[B4-jintelligence-13-00065] Breit Moritz, Scherrer Veronika, Preckel Francis (2024). How useful are specific cognitive ability scores? An investigation of their stability and incremental validity beyond general intelligence. Intelligence.

[B5-jintelligence-13-00065] Caemmerer Jacqueline M., Reynolds Matthew R., Keith Timothy Z. (2023). Beyond individual tests: Youth’s cognitive abilities on their math and writing skills. Learning and Individual Differences.

[B6-jintelligence-13-00065] Caemmerer Jacqueline M., Young Stephanie R., Maddocks Danika, Charamut Natalie R., Blemahdoo Eunice (2024). Predicting achievement from WISC-V composites: Do cognitive-achievement relations vary based on general intelligence?. Journal of Psychoeducational Assessment.

[B7-jintelligence-13-00065] Carrigan Joseph E. (2023). Are the Effects of *g* on Achievement Smaller at Higher Ability Levels?. Ph.D. dissertation.

[B8-jintelligence-13-00065] Carroll John B. (1993). Human Cognitive Abilities: A Survey of Factor-Analytic Studies.

[B9-jintelligence-13-00065] Chu Felicia, van Marle Kristy, Geary David C. (2016). Predicting children’s reading and mathematics achievement from early quantitative knowledge and domain-general cognitive abilities. Frontiers in Psychology.

[B10-jintelligence-13-00065] Cole David A., Maxwell Scott E. (2003). Testing mediational models with longitudinal data: Questions and tips in the use of structural equation modeling. Journal of Abnormal Psychology.

[B11-jintelligence-13-00065] Cormier Damien C., Bulut Okan, McGrew Kevin S., Singh Deepak (2017). Exploring the relations between Cattell–Horn–Carroll (CHC) cognitive abilities and mathematics achievement. Applied Cognitive Psychology.

[B12-jintelligence-13-00065] Coyle Thomas, Snyder Anissa, Pillow David, Kochunov Peter (2011). SAT predicts GPA better for high ability subjects: Implications for Spearman’s Law of Diminishing Returns. Personality and Individual Differences.

[B13-jintelligence-13-00065] Curran Patrick J., West Stephen G., Finch John F. (1996). The robustness of test statistics to nonnormality and specification error in confirmatory factor analysis. Psychological Methods.

[B14-jintelligence-13-00065] Deary Ian J., Strand Steve, Smith Pauline, Fernandes Cres (2007). Intelligence and educational achievement. Intelligence.

[B15-jintelligence-13-00065] Decker Scott, Roberts Alycia (2015). Specific cognitive predictors of early math problem solving. Psychology in the Schools.

[B16-jintelligence-13-00065] Detterman Douglas, Daniel Mark H. (1989). Correlations of mental tests with each other and with cognitive variables are highest for low IQ groups. Intelligence.

[B17-jintelligence-13-00065] Duckworth Kathryn, Duncan Greg J., Kokko Katja, Lyyra Anna-Liisa, Metzger Molly, Simonton Sharon (2012). The Relative Importance of Adolescent Skills and Behaviors for Adult Earnings: A Cross-National Study.

[B18-jintelligence-13-00065] Enders Craig K. (2022). Applied Missing Data Analysis.

[B19-jintelligence-13-00065] Fletcher Jack, Lyon Reid, Fuchs Lynn, Barnes Marcia (2019). Learning Disabilities: From Identification to Intervention.

[B20-jintelligence-13-00065] Floyd Randy G., Evans Jeffrey J., McGrew Kevin S. (2003). Relations between measures of Cattell-Horn-Carroll (CHC) cognitive abilities and mathematics achievement across the school-age years. Psychology in the Schools.

[B21-jintelligence-13-00065] Floyd Randy G., Meisinger Elizabeth, Gregg Noel, Keith Timothy Z. (2012). An explanation of reading comprehension across development using models from Cattell–Horn–Carroll theory: Support for integrative models of reading. Psychology in the Schools.

[B22-jintelligence-13-00065] Gottfredson Linda S. (1997). Why *g* matters: The complexity of everyday life. Intelligence.

[B23-jintelligence-13-00065] Hajovsky Daniel B., Niileksela Christopher R., Olsen Sunny C., Sekula Morgan K. (2023). Do cognitive–achievement relations vary by general ability level?. Journal of Intelligence.

[B24-jintelligence-13-00065] Hajovsky Daniel B., Villeneuve Ethan F., Schneider William Joel, Caemmerer Jacqueline M. (2020). An alternative approach to cognitive and achievement relations research: An introduction to quantile regression. Journal of Pediatric Neuropsychology.

[B25-jintelligence-13-00065] Hajovsky Daniel B., Reynolds Matthew R., Floyd Randy G., Turek Joshua, Keith Timothy Z. (2014). A multigroup investigation of latent cognitive abilities and reading achievement relations. School Psychology Review.

[B26-jintelligence-13-00065] Haberstroh Stefan, Schulte-Körne Gerd (2022). The cognitive profile of math difficulties: A meta-analysis based on clinical criteria. Frontiers in Psychology.

[B27-jintelligence-13-00065] Henry Lucy, Winfield Jill (2010). Working memory and educational achievement in children with intellectual disabilities. Journal of Intellectual Disability Research: Journal of Intellectual Disability Research.

[B28-jintelligence-13-00065] Jansen Amanda, Cooper Brandy, Vascellaro Stefanie, Wandless Philip (2016). Rough-Draft Talk in Mathematics Classrooms. Mathematics Teaching in the Middle School.

[B29-jintelligence-13-00065] Kane Harrison D., Oakland Thomas D., Brand Christopher R. (2006). Differentiation at higher levels of cognitive ability: Evidence from the United States. The Journal of Genetic Psychology.

[B30-jintelligence-13-00065] Kaufman Scott B., Reynolds Matthew R., Liu Xin, Kaufman Alan S., McGrew Kevin S. (2012). Are cognitive *g* and academic achievement *g* one and the same *g*? An exploration on the Woodcock-Johnson and Kaufman tests. Intelligence.

[B31-jintelligence-13-00065] Kuncel Nathan R., Hezlett Sarah A. (2010). Fact and fiction in cognitive ability testing for admissions and hiring decisions. Current Directions in Psychological Science.

[B32-jintelligence-13-00065] Lewis Katherine E., Fisher Marie B. (2016). Taking stock of 40 years of research on mathematical learning disability: Methodological issues and future directions. Journal for Research in Mathematics Education.

[B33-jintelligence-13-00065] McGee Rob, Prior Margot, Williams Sheila, Smart Diana, Sanson Anne (2002). The long-term significance of teacher-rated hyperactivity and reading ability in childhood: Findings from two longitudinal studies. Journal of Child Psychology and Psychiatry.

[B34-jintelligence-13-00065] McGill Ryan J. (2015). Spearman’s Law of Diminishing Returns (SLODR): Examining effects at the level of prediction. Journal of Psychology and Behavioral Science.

[B35-jintelligence-13-00065] McGrew Kevin S., Wendling Barbara (2010). Cattell–Horn–Carroll cognitive-achievement relations: What we have learned from the past 20 years of research. Psychology in the Schools.

[B36-jintelligence-13-00065] McGrew Kevin S., Schrank Fredrick A., Woodcock Richard W. (2007). Technical Manual. Woodcock-Johnson III Normative Update.

[B37-jintelligence-13-00065] McGrew Kevin S., LaForte Erica M., Schrank Fredrick A. (2014). Technical Manual. Woodcock-Johnson IV.

[B38-jintelligence-13-00065] McLarnon Matthew, Goffin Richard, Rothstein Mitchell G. (2018). Differentiation of cognitive abilities and the medical college admission test. Personality and Individual Differences.

[B39-jintelligence-13-00065] Murray Aja L., Dixon Hayley, Johnson Wendy (2013). Spearman’s law of diminishing returns: A statistical artifact?. Intelligence.

[B40-jintelligence-13-00065] Muthén Linda K., Muthén Bengt O. (1998–2015). Mplus User’s Guide.

[B41-jintelligence-13-00065] Niileksela Christopher R., Reynolds Matthew R., Keith Timothy Z., McGrew Kevin S., Flanagan Dawn P., Alfonso Vincent C. (2016). A special validity study of the WJ IV: Acting on evidence for specific abilities. WJ IV Clinical Use and Interpretation: Scientist-Practitioner Perspectives.

[B42-jintelligence-13-00065] Peng Peng, Kievit Rogier A. (2020). The development of academic achievement and cognitive abilities: A bidirectional perspective. Child Development Perspectives.

[B43-jintelligence-13-00065] Peng Peng, Wang Cuicui, Namkung Jessica (2018). Understanding the cognition related to mathematics difficulties: A meta-analysis on the cognitive deficit profiles and the bottleneck theory. Review of Educational Research.

[B44-jintelligence-13-00065] Preacher Kristopher J. (2015). Advances in mediation analysis: A survey and synthesis of new developments. Annual Review of Psychology.

[B45-jintelligence-13-00065] Reyna Valerie F., Brainerd Charles J. (2007). The importance of mathematics in health and human judgment: Numeracy, risk communication, and medical decision making. Learning and Individual Differences.

[B46-jintelligence-13-00065] Reynolds Matthew R., Hajovsky Daniel B., Niileksela Christopher R., Keith Timothy Z. (2011). Spearman’s law of diminishing returns and the DAS-II: Do *g* effects on subtest scores depend on the level of *g*?. School Psychology Quarterly.

[B47-jintelligence-13-00065] Reynolds Matthew R., Keith Timothy Z., Beretvas S. Natasha (2010). Use of factor mixture modeling to capture Spearman’s law of diminishing returns. Intelligence.

[B48-jintelligence-13-00065] Rinaldi Luca, Karmiloff-Smith Annette (2017). Intelligence as a developing function: A neuroconstructivist approach. Journal of Intelligence.

[B49-jintelligence-13-00065] Schermelleh-Engel Karin, Moosbrugger Helfried, Müller Hans (2003). Evaluating the fit of structural equation models: Tests of significance and descriptive goodness-of-fit measures. Methods of Psychological Research Online.

[B50-jintelligence-13-00065] Schmitt Sara A., Geldhof G. John, Purpura David J., Duncan Robert, McClelland Morgan M. (2017). Examining the relations between executive function, math, and literacy during the transition to kindergarten: A multianalytic approach. Journal of Educational Psychology.

[B51-jintelligence-13-00065] Schrank Fredrick A., McGrew Kevin S., Mather Nancy (2014). Woodcock-Johnson IV.

[B52-jintelligence-13-00065] Schneider W. Joel, McGrew Kevin S. (2018). The Cattell-Horn-Carroll theory of intelligence. Contemporary Intellectual Assessment.

[B53-jintelligence-13-00065] Spearman Charles (1927). The Abilities of Man: Their Nature and Measurement.

[B54-jintelligence-13-00065] Sterner Gorel, Wolff Ulrika, Helenius Ola (2020). Reasoning about representations: Effects of an early math intervention. Scandinavian Journal of Educational Research.

[B55-jintelligence-13-00065] Stockard Jean, Wood Timothy W., Coughlin Cristy, Khoury Caitlin R. (2018). The effectiveness of direct instruction curricula: A meta-analysis of a half century of research. Review of Educational Research.

[B56-jintelligence-13-00065] Taub Gordon E., Keith Timothy Z., Floyd Randy G., McGrew Kevin S. (2008). Effects of general and broad cognitive abilities on mathematics achievement. School Psychology Quarterly.

[B57-jintelligence-13-00065] Thurber Robin S., Shinn Mark R., Smolkowski Keith (2002). What is Measured in Mathematics Tests? Construct Validity of Curriculum-Based Mathematics Measures. School Psychology Review.

[B58-jintelligence-13-00065] Thurstone Louis L. (1938). Primary Mental Abilities.

[B59-jintelligence-13-00065] Träff Ulf, Olsson Linda, Östergren Rickard, Skagerlund Kenny (2020). Development of early domain-specific and domain-general cognitive precursors of high and low math achievers in grade 6. Child Neuropsychology.

[B60-jintelligence-13-00065] van der Maas Han, Dolan Conor, Grasman Raoul, Wicherts Jelte (2006). A dynamical model of general intelligence: The positive manifold of intelligence by mutualism. Psychological Review.

[B61-jintelligence-13-00065] Viesel-Nordmeyer Nurit, Reuber Julia, Kuhn Jorg-Tobias, Mill Kristina, Holling Heinz, Dobel Christian (2023). Cognitive profiles of children with isolated and comorbid learning difficulties in reading and math: A meta-analysis. Educational Psychology Review.

[B62-jintelligence-13-00065] Villeneuve Ethan F., Hajovsky Daniel B., Mason Benjamin A., Lewno Brittany M. (2019). Cognitive ability and math computation developmental relations with math problem solving: An integrated, multigroup approach. School Psychology.

[B63-jintelligence-13-00065] Woodcock Richard W., McGrew Kevin S., Schrank Fredrick A., Mather Nancy (2001, 2007). Woodcock-Johnson III Normative Update.

[B64-jintelligence-13-00065] Wrulich Marius, Brunner Martin, Stadler Gertraud, Schalke Daniela, Keller Ulrich, Martin Romain (2014). Forty years on: Childhood intelligence predicts health in middle adulthood. Health Psychology.

[B65-jintelligence-13-00065] Zaboski Brian A., Kranzler John H., Gage Nicholas A. (2018). Meta-analysis of the relationship between academic achievement and broad abilities of the Cattell-Horn-Carroll theory. Journal of School Psychology.

[B66-jintelligence-13-00065] Zhang Jingyuan, Martella Ronald C., Kang Sungwoo, Yenioglu Busra Yilmaz (2023). Response to intervention (RTI)/multi-tiered systems of support (MTSS): A nationwide analysis. Journal of Educational Leadership and Policy Studies.

[B67-jintelligence-13-00065] Zhang Zheng, Peng Peng (2023). Co-development among reading, math, science, and verbal working memory in the elementary stage. Child Development.

